# Association Between Left Ventricular Geometry and Renal Outcomes in Patients With Chronic Kidney Disease: Findings From Korean Cohort Study for Outcomes in Patients With Chronic Kidney Disease Study

**DOI:** 10.3389/fcvm.2022.848692

**Published:** 2022-04-18

**Authors:** Sang Heon Suh, Tae Ryom Oh, Hong Sang Choi, Chang Seong Kim, Eun Hui Bae, Kook-Hwan Oh, Joongyub Lee, Ji Yong Jung, Kyu-Beck Lee, Seong Kwon Ma, Soo Wan Kim

**Affiliations:** ^1^Department of Internal Medicine, Chonnam National University Medical School and Chonnam National University Hospital, Gwangju, South Korea; ^2^Department of Internal Medicine, Seoul National University Hospital, Seoul, South Korea; ^3^Department of Prevention and Management, School of Medicine, Inha University, Incheon, South Korea; ^4^Division of Nephrology, Department of Internal Medicine, Gachon University of Gil Medical Center, Incheon, South Korea; ^5^Department of Internal Medicine, School of Medicine, Kangbuk Samsung Hospital, Sungkyunkwan University Seoul, Seoul, South Korea

**Keywords:** chronic kidney disease, left ventricular geometry, left ventricular hypertrophy, relative wall thickness, renal outcome, all-cause mortality

## Abstract

**Background:**

The impact of left ventricular (LV) geometry on the renal outcomes in patients with chronic kidney disease (CKD) has not been established yet. We aimed to investigate the association of LV geometry with renal outcomes and all-cause mortality in patients with pre-dialysis CKD.

**Methods:**

A total of 2,144 subjects from the Korean Cohort Study for Outcome in Patients With Chronic Kidney Disease (KNOW-CKD) were categorized by LV geometry, which was defined by LV mass index and relative wall thickness [normal geometry, concentric remodeling, eccentric hypertrophy (eLVH), and concentric hypertrophy (cLVH)]. Study outcomes were composite renal events [decline of kidney function (the first occurrence of > 50% decline of eGFR or doubling of serum creatinine from the baseline) and onset of ESRD (initiation of dialysis or kidney transplantation) during follow-up periods)] and all-cause mortality.

**Results:**

Cox regression analysis revealed that eLVH [adjusted hazard ratio (HR) 1.498, 95% confidence interval (CI) 1.197–1.873] and cLVH (adjusted HR 1.289, 95% CI 1.011–1.643) were associated with increased risk of composite renal events, whereas concentric remodeling (adjusted HR 1.881, 95% CI 1.135–3.118) and cLVH (adjusted HR 2.216, 95% CI 1.341–3.664) were associated with increased risk of all-cause mortality. Sensitivity analyses confirmed that concentric remodeling (adjusted HR 1.993, 95% CI 1.197–3.368) and eLVH (adjusted HR 1.588, 95% CI 1.261–2.001) are independently associated with all-cause mortality and composite renal events, respectively.

**Conclusion:**

In conclusion, we report that LV geometry is significantly associated with adverse renal outcomes and all-cause mortality in patients with pre-dialysis CKD. Echocardiographic determination of LV geometry may help the early identification for the patients with high risk of CKD progression.

## Introduction

Structural remodeling of heart predicts adverse cardiovascular (CV) outcomes, which is best illustrated by left ventricular hypertrophy (LVH). LVH is a surrogate of CV events, such as heart failure, ischemic heart disease, and stroke (1), as the reversal of LVH by antihypertensive treatment has been associated with the reduction of the risk for subsequent CV events (2–4). Relative wall thickness (RWT), together with left ventricular (LV) mass, is commonly obtained echocardiographic parameter to describe the shape of heart (5), and also provides additional and independent prognostic impacts (6, 7). Accordingly, LV geometry has been classified into four categories by left ventricular mass index (LVMI) and RWT (8): normal geometry, concentric remodeling, eccentric hypertrophy (eLVH), and concentric hypertrophy (cLVH).

Kidney targets heart, as cardiac remodeling begins from the early stages of chronic kidney disease (CKD) (9, 10). The multifactorial mechanism of LVH involves increased afterload (11, 12), intravascular volume expansion (13), and anemia (14), all of which are closely related to CKD. The presence of arteriovenous fistula further accelerates cardiac remodeling in patients with end-stage renal disease (ESRD) (15). The prevalence of LVH in this population is estimated to be up to about 30% in individuals with an estimated glomerular filtration rate (eGFR) > 30 ml/min/1.73 m^2^, and it increases to 60–75% prior to initiation of dialysis (16). A previous study reported that the prevalence of normal LV geometry was less than 10% among the subjects with eGFR < 30 ml/min/1.73 m^2^ (9). Inversely, LVH increased the risk of ESRD among hypertensive patients (17), and is significantly associated with adverse CV events and all-cause mortality especially in patients on dialysis (18–20). Indeed, a study reported that LVH was associated with almost twofold increase of the risk for sudden cardiac death in patients on hemodialysis (20). Yet, the prognostic significance of LVH in patients with pre-dialysis CKD has been less clearly documented (21). Moreover, the independent association of RWT on renal outcomes in patients with pre-dialysis CKD has been never reported.

Focusing on the impact of cardiac remodeling on CKD progression, we here investigated the association of LV geometry with renal outcomes and all-cause mortality in patients with pre-dialysis CKD. In addition, the association of LVH or RWT with the outcomes was separately analyzed to address their independent role as a prognostic predictor. Finally, we conducted a series of subgroup analyses to examine whether the association of LV geometry with the outcomes might be modified by clinical contexts.

## Materials and Methods

### Study Designs

**FIGURE 1:**
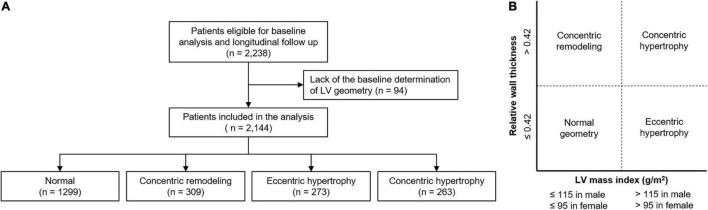
Flow diagram of the study participants. Flow diagram of the study participants **(A)** and definition of abnormal LV geometry **(B)** are depicted. LV, left ventricle.

The Korean Cohort Study for Outcomes in Patients With Chronic Kidney Disease (KNOW-CKD) is a nationwide prospective cohort study involving 9 tertiary-care general hospitals in Korea (NCT01630486)^1^ (22). Korean patients with CKD from stage 1 to pre-dialysis stage 5, who voluntarily provided informed consent were enrolled from 2011 through 2016. The study was conducted in accordance with the principles of the Declaration of Helsinki. The study protocol was approved by the institutional review boards of participating centers, including at Seoul National University Hospital, Yonsei University Severance Hospital, Kangbuk Samsung Medical Center, Seoul St. Mary’s Hospital, Gil Hospital, Eulji General Hospital, Chonnam National University Hospital, and Busan Paik Hospital. All participants had been under close observation, and participants who experienced study outcomes were reported by each participating center. Among 2,238 who were longitudinally followed up, excluding those lacking the baseline determination of LV geometry, a total of 2,144 subjects were finally included for the analyses (Figure 1A). The study observation period ended on March 31, 2020. The median follow-up duration was 5.999 years.

### Data Collection From Participants

Demographic information was collected from all eligible participants, including age, gender, comorbid conditions, primary renal disease, smoking history, and medication history (angiotensin-converting enzyme inhibitor/angiotensin II receptor blockers (ACEi/ARBs), diuretics, number of anti-HTN drugs, statins). Trained staff members measured the height, weight, and waist circumference (WC) of study participants. Body mass index (BMI) was calculated as weight divided by the height squared. Systolic and diastolic blood pressures (SBP and DBP) were measured by an electronic sphygmomanometer after seated rest for 5 min. Venous samples were collected following overnight fasting, to determine hemoglobin, albumin, total cholesterol, low density lipoprotein cholesterol, high density lipoprotein cholesterol (HDL-C), triglyceride (TG), fasting glucose, high-sensitivity C-reactive protein (hs-CRP), 25-hydroxyvitamin D [25(OH) vitamin D] and creatinine levels at the baseline. eGFR was calculated by CKD Epidemiology Collaboration equation (23). Urine albumin-to-creatinine ratio (ACR) was measured in random, preferably second-voided, spot urine samples.

### Echocardiographic Data Collection

Complete two-dimensional M-mode and Doppler studies were performed *via* standard approaches by cardiologists at the participating hospitals who were blinded to the clinical data. M-mode examination was performed according to American Society of Echocardiography guidelines (8). The recorded echocardiographic data were the ratio of the early transmitral blood flow velocity to early diastolic velocity of the mitral annulus, left ventricular ejection fraction (LVEF), left atrial diameter, regional wall motion abnormality, valve calcification, LV posterior wall thickness, inter-ventricular septum thickness, LV end diastolic diameter, and LV end systolic diameter. LV mass was determined using the Devereux formula (8). LVMI was calculated by normalizing LV mass to height^2^ (g/m^2^). LVH was defined as LVMI > 115 g/m^2^ in men and > 95 g/m^2^ in women (7, 8). RWT was calculated as (2 × posterior wall thickness)/LV end diastolic diameter. RWT > 0.42 was defined as increased (7, 8, 10). LV geometry was determined by LVH and RWT: normal (no LVH and normal RWT), concentric remodeling (no LVH and increased RWT), eLVH (LVH and normal RWT), and cLVH (LVH and increased RWT) (Figure 1B).

### Study Outcomes

The primary outcomes of interest were composite renal events and all-cause mortality. Composite renal events included decline of kidney function (the first occurrence of > 50% decline of eGFR or doubling of serum creatinine from the baseline) and onset of ESRD (initiation of dialysis or kidney transplantation) during follow-up periods. The secondary outcomes were decline of kidney function and onset of ESRD.

### Statistical Analysis

Continuous variables were expressed as mean ± standard deviation or median [interquartile range]. Categorical variables were expressed as number of participants and percentage. To compare the baseline characteristics according to LV geometry, one-way analysis of variance and χ^2^-test were used for continuous and categorical variates, respectively. The participants with any missing data were excluded for further analyses. To evaluate the association between LV diastolic dysfunction and study outcomes, Cox proportional hazard regression models were analyzed. Patients lost to follow-up were censored at the date of the last visit. Models were constructed after adjusting for the following variables. Model 1 represents crude hazard ratios (HRs). Model 2 was adjusted for age, sex, Charlson comorbidity index, primary renal disease, smoking history, medication (ACEi/ARBs, diuretics, number of antihypertensive drugs, statins), BMI, and SBP. Model 3 was further adjusted for hemoglobin, albumin, fasting glucose, HDL-C, TG, 25(OH) vitamin D, hs-CRP, eGFR and spot urine ACR. Model 4 was additionally adjusted for LVEF at the baseline. The results of Cox proportional hazard models were presented as HRs and 95% confidence intervals (CIs). Restricted cubic splines were used to visualize the association between LVMI or RWT as a continuous variable and HRs for study outcomes. To validate our findings, we performed sensitivity analyses. First, we excluded the subjects with LEVE < 50% to demonstrate that the association between LV geometry and study outcomes is independent of LV systolic dysfunction. Second, we excluded the subjects with increased RWT to figure out an independent role of LVH in the study outcomes. Third, we conversely excluded the subjects with LVH to unveil an independent role of RWT in the study outcomes. To examine whether the association of LV geometry with the outcomes might be modified by clinical contexts, we conducted pre-specified subgroup analyses. Subgroups were defined by age (< 60 vs. (vs.) ≥ 60 years), sex (male vs. female), BMI (< 23 vs. ≥ 23 kg/m^2^), eGFR (< 45 vs. ≥ 45 mL/min/1.73 m^2^), and spot urine ACR (< 300 vs. ≥ 300 mg/gCr). Two-sided *P*-values < 0.05 were considered statistically significant. Statistical analysis was performed using SPSS for Windows version 22.0 (IBM Corp., Armonk, NY) and R (version 4.1.1; R project for Statistical Computing, Vienna, Austria).

## Results

### Baseline Characteristics

To describe the baseline characteristics, study participants were categorized by LV geometry (Table 1). Whereas the follow-up duration was significantly shortened in the subjects with cLVH, the mean age was highest in the subjects with cLVH. The proportion of male sex was relatively lower in the subjects with eLVH and cLVH, than in those with normal geometry and concentric remodeling. The proportion of Charlson comorbidity index ≥ 4 was highest in the subjects with cLVH. The proportion of DM and HTN as primary renal diseases was relatively higher in the subjects with eLVH and cLVH, than in those with normal geometry and concentric remodeling. The proportion of smoking history was lower in the subjects with eLVH and cLVH. The proportions of diuretic use, medication of no less than three anti-HTN drugs, statin medication were higher in the subjects with eLVH and cLVH. BMI, WC, SBP were highest in the subjects with cLVH. While hemoglobin level was lowest in the subjects with eLVH, albumin, HDL-C, 25(OH) vitamin D levels were lowest in the subjects with cLVH. Fasting glucose and TG levels were highest in the subjects with cLVH. Spot urine ACR and eGFR were highest and lowest in the subjects with cLVH, respectively. Accordingly, the proportion of relatively advance CKD was higher in the subjects with cLVH. The echocardiographic findings of study participants by LV geometry is summarized in Supplementary Table 1. Besides the structural measurement directly related to LV geometry, other parameters demonstrated significant differences by LV geometry. The ratio of the early transmitral blood flow velocity to early diastolic velocity of the mitral annulus and left atrial diameter were significantly higher in the subjects with eLVH and cLVH. The proportions of regional wall motion abnormality and valve calcification were also significantly higher in the subjects with eLVH and cLVH. LVEF was lowest in the subjects with eLVH.

**TABLE 1:** Baseline characteristics of study participants by LV geometry.

	LV geometry				
	
	Normal	Concentric remodeling	Eccentric hypertrophy	Concentric hypertrophy	*P*-value
Follow-up duration (year)	5.758 ± 2.071	5.362 ± 2.274	5.577 ± 2.424	5.002 ± 2.324	<0.001
Age (year)	51.186 ± 12.354	55.994 ± 11.845	56.967 ± 10.524	59.172 ± 10.745	<0.001
Male	827 (63.7)	222 (71.8)	109 (40.1)	152 (58.0)	<0.001
Charlson comorbidity index					<0.001
0–3	1,026 (79.0)	199 (64.4)	169 (61.9)	145 (55.1)	
4–5	259 (19.9)	103 (33.3)	97 (35.5)	110 (41.8)	
≥ 6	14 (1.1)	7 (2.3)	7 (2.6)	8 (3.0)	
Primary renal disease					<0.001
DM	251 (19.4)	89 (28.8)	91 (33.5)	104 (39.7)	
HTN	229 (17.7)	70 (22.7)	56 (20.6)	66 (25.2)	
GN	480 (37.0)	81 (26.2)	68 (25.0)	52 (19.8)	
TID	6 (0.5)	6 (1.9)	1 (0.4)	0 (0.0)	
PKD	253 (19.5)	42 (13.6)	32 (11.8)	21 (8.0)	
Others	78 (6.0)	21 (6.8)	24 (8.8)	19 (7.3)	
Smoking history	604 (46.5)	177 (57.3)	94 (34.6)	125 (47.7)	<0.001
**Medication**					
ACEi/ARBs	1,113 (85.7)	263 (85.1)	223 (82.0)	233 (88.9)	0.150
Diuretics	320 (24.7)	113 (36.6)	116 (42.6)	123 (46.9)	<0.001
Number of anti-HTN drugs ≥ 3	306 (23.6)	84 (27.2)	100 (36.8)	129 (49.2)	<0.001
Statins	618 (47.6)	179 (57.9)	149 (54.8)	161 (61.5)	<0.001
BMI (kg/m^2^)	24.250 ± 3.375	24.907 ± 3.442	24.815 ± 3.188	25.530 ± 3.518	<0.001
WC (cm)	86.374 ± 9.698	89.122 ± 9.098	87.334 ± 9.455	89.934 ± 9.746	<0.001
SBP (mmHg)	126.116 ± 15.455	127.188 ± 15.413	130.801 ± 16.680	133.667 ± 18.643	<0.001
DBP (mmHg)	76.950 ± 10.687	77.074 ± 10.743	76.886 ± 11.816	77.160 ± 12.737	0.989
Laboratory findings					
Hemoglobin (g/dL)	13.088 ± 1.943	13.226 ± 2.082	11.926 ± 1.862	12.170 ± 2.049	<0.001
Albumin (g/dL)	4.209 ± 0.400	4.223 ± 0.452	4.069 ± 0.418	4.089 ± 0.473	<0.001
Total cholesterol (mg/dL)	174.476 ± 37.783	173.498 ± 42.810	172.221 ± 36.002	173.794 ± 38.785	0.847
HDL-C (mg/dL)	50.326 ± 15.780	47.114 ± 13.302	48.806 ± 15.553	46.568 ± 15.239	<0.001
LDL-C (mg/dL)	97.535 ± 32.154	97.096 ± 34.541	93.745 ± 27.798	95.448 ± 30.148	0.302
TG (mg/dL)	152.089 ± 92.902	164.957 ± 104.052	161.943 ± 104.509	172.921 ± 112.942	0.013
Fasting glucose (mg/dL)	107.970 ± 34.511	114.808 ± 40.829	111.940 ± 42.849	116.862 ± 51.477	0.003
25 (OH) Vitamin D (ng/mL)	17.992 ± 7.385	18.021 ± 7.925	17.617 ± 8.004	16.663 ± 9.624	0.191
hsCRP (mg/dL)	0.580 [0.200, 1.500]	0.7400 [0.300, 1.820]	0.800 [0.300, 2.000]	0.800 [0.400, 1.800]	0.838
Spot urine ACR (mg/gCr)	281.014 [53.832, 817.945]	312.492 [71.983, 1011.896]	530.272 [183.957, 1600.001]	775.0.34 [170.073, 1736.877]	<0.001
eGFR (mL/min./1.73 m^2^)	55.167 ± 31.219	48.714 ± 26.283	41.917 ± 27.421	39.018 ± 26.823	<0.001
CKD stages					<0.001
Stage 1	269 (20.7)	35 (11.3)	26 (9.6)	19 (7.3)	
Stage 2	275 (21.2)	60 (19.4)	39 (14.3)	33 (12.6)	
Stage 3a	219 (16.9)	60 (19.4)	34 (12.5)	35 (13.4)	
Stage 3b	257 (19.8)	80 (25.9)	59 (21.7)	57 (21.8)	
Stage 4	220 (16.9)	59 (19.1)	91 (33.5)	85 (32.4)	
Stage 5	58 (4.5)	15 (4.9)	23 (8.5)	33 (12.6)	

*Values for categorical variables are given as number (percentage); values for continuous variables, as mean ± standard deviation or median [interquartile range]. ACEi, angiotensin converting enzyme inhibitor; ACR, albumin-to-creatinine ratio; ARB, angiotensin receptor blocker; BMI, body mass index; CKD, chronic kidney disease; Cr, creatinine; DBP, diastolic blood pressure; DM, diabetes mellitus; eGFR, estimated glomerular filtration rate; GN, glomerulonephritis; HDL-C, high density lipoprotein cholesterol; hsCRP, high-sensitivity C-reactive protein; HTN, hypertension; LDC-C, low density lipoprotein cholesterol; PKD, polycystic kidney disease; SBP, systolic blood pressure; TG, triglyceride; TID, tubulointerstitial disease; WC, waist circumference.*

### Association of Left Ventricular Geometry With Adverse Renal Outcome and All-Cause Mortality in Chronic Kidney Disease

**FIGURE 2:**
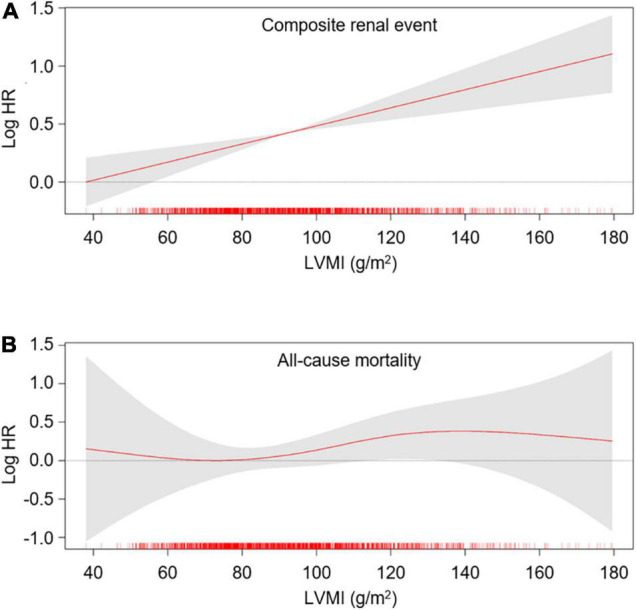
Restricted cubic spline of LVMI on primary outcomes. Adjusted HRs of LVMI as a continuous variable for composite renal events **(A)** and all-cause mortality **(B)** is depicted. The model was adjusted for age, sex, Charlson comorbidity index, primary renal disease, smoking history, medication (ACEi/ARBs, diuretics, number of anti-HTN drugs, statins), BMI, SBP, hemoglobin, albumin, fasting glucose, HDL-C, TG, 25(OH) vitamin D, hs-CRP, GFR, spot urine ACR and EF at the baseline. HR, hazard ratio; LVMI, left ventricular mass index.

**FIGURE 3:**
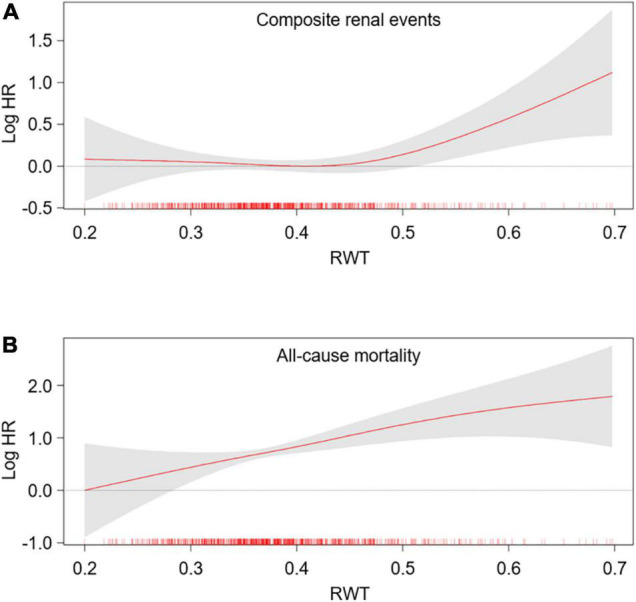
Restricted cubic spline of RWT on primary outcomes. Adjusted HRs of RWT as a continuous variable for composite renal events **(A)** and all-cause mortality **(B)** is depicted. The model was adjusted for age, sex, Charlson comorbidity index, primary renal disease, smoking history, medication (ACEi/ARBs, diuretics, number of anti-HTN drugs, statins), BMI, SBP, hemoglobin, albumin, fasting glucose, HDL-C, TG, 25(OH) vitamin D, hs-CRP, GFR, spot urine ACR and EF at the baseline. HR, hazard ratio; RWT, relative wall thickness.

To define the association of LV geometry with study outcomes, Cox regression models were analyzed (Table 2). Compared to normal geometry, eLVH (adjusted HR 1.498, 95% CI 1.197–1.873) and cLVH (adjusted HR 1.289, 95% CI 1.011–1.643) were associated with increased risk of composite renal events, whereas concentric remodeling was not significantly associated with the risk of composite renal events. In contrast, concentric remodeling (adjusted HR 1.881, 95% CI 1.135–3.118) and cLVH (adjusted HR 2.216, 95% CI 1.341–3.664) were associated with increased risk of all-cause mortality, whereas eLVH was not significantly associated with the risk of all-cause mortality. In the analysis of secondary outcomes, only eLVH was significantly associated with both decline of kidney function (adjusted HR 1.535, 95% CI 1.171–2.013) and onset of ESRD (adjusted HR 1.390, 95% CI 1.072–1.802) (Supplementary Table 2). As the results suggested that LVH and concentric geometry (i.e., increased RWT, including concentric remodeling and cLVH) were associated with adverse renal outcome and all-cause mortality, respectively, we separately analyzed the association of LVH or RWT with the outcomes to address their independent role as a prognostic predictor. LVH was associated with increased risk of composite renal events (adjusted HR 1.391, 95% CI 1.161–1.667), but was not significantly associated with all-cause mortality (Supplementary Table 3). Restricted cubic splines visualized stringent linear correlation of LVMI with the risk of composite renal events, but not with all-cause mortality (Figure 2). On the other hand, concentric geometry was associated with all-cause mortality (adjusted HR 2.038, 95% CI 1.391–2.984), but was not significantly associated with the risk of composite renal events (Table 3). Restricted cubic splines demonstrated stringent linear correlation of RWT with all-cause mortality, while the correlation between RWT and the risk of composite renal events was evident only among the subjects with very high RWT (Figure 3).

**TABLE 2:** Cox regression analysis of LV geometry for primary outcomes.

	LV geometry	Events, *n* (%)	Model 1		Model 2		Model 3		Model 4	
						
			HR (95% CIs)	*P*-value	HR (95% CIs)	*P*-value	HR (95% CIs)	*P*-value	HR (95% CIs)	*P*-value
Composite renal event	Normal	402 (31.0)	Reference		Reference		Reference		Reference	
	Concentric remodeling	94 (30.4)	1.071 (0.847, 1.354)	0.567	0.911 (0.724, 1.145)	0.423	1.024 (0.805, 1.303)	0.847	1.022 (0.804, 1.301)	0.856
	Eccentric hypertrophy	133 (48.9)	1.933 (1.570, 2.380)	<0.001	1.528 (1.235, 1.89)	<0.001	1.473 (1.179, 1.841)	<0.001	1.498 (1.197, 1.873)	<0.001
	Concentric hypertrophy	112 (42.7)	1.984 (1.585, 2.484)	<0.001	0.98 (0.974, 0.987)	<0.001	1.281 (1.005, 1.634)	0.046	1.289 (1.011, 1.643)	0.041
All-cause mortality	Normal	50 (3.9)	Reference		Reference		Reference		Reference	
	Concentric remodeling	29 (9.4)	2.506 (1.549, 4.054)	<0.001	1.88 (1.176, 3.007)	0.008	1.852 (1.118, 3.067)	0.0167	1.881 (1.135, 3.118)	0.014
	Eccentric hypertrophy	18 (6.6)	1.742 (0.986, 3.077)	0.056	1.192 (0.680, 2.089)	0.539	1.097 (0.604, 1.994)	0.7607	1.015 (0.554, 1.86)	0.962
	Concentric hypertrophy	38 (14.5)	4.706 (3.030, 7.308)	<0.001	2.574 (1.632, 4.061)	<0.001	2.194 (1.328, 3.623)	0.0021	2.216 (1.341, 3.664)	0.002

*Model 1, unadjusted model. Model 2, model 1 + adjusted for age, sex, Charlson comorbidity index, primary renal disease, smoking history, medication (ACEi/ARBs, diuretics, number of anti-HTN drugs, statins), BMI, and SBP. Model 3, model 2 + adjusted for hemoglobin, albumin, fasting glucose, HDL-C, TG, 25(OH) vitamin D, hs-CRP, GFR and spot urine ACR. Model 4, model 3 + adjusted for EF at the baseline. CI, confidence interval; HR, hazard ratio.*

**TABLE 3:** Cox regression analysis of RWT for primary outcomes.

	RWT	Events, *n* (%)	Model 1		Model 2		Model 3		Model 4	
						
			HR (95% CIs)	*P*-value	HR (95% CIs)	*P*-value	HR (95% CIs)	*P*-value	HR (95% CIs)	*P*-value
Composite renal event	Normal	535 (34.1)	Reference		Reference		Reference		Reference	
	Increased	206 (36.1)	1.244 (1.049, 1.475)	0.012	1.062 (0.900, 1.253)	0.479	1.042 (0.872, 1.245)	0.651	1.041 (0.871, 1.244)	0.660
All-cause mortality	Normal	68 (4.3)	Reference		Reference		Reference		Reference	
	Increased	67 (11.7)	3.048 (2.140, 4.342)	<0.001	2.108 (1.485, 2.992)	<0.001	1.969 (1.349, 2.874)	<0.001	2.038 (1.391, 2.984)	<0.001

*Model 1, unadjusted model. Model 2, model 1 + adjusted for age, sex, Charlson comorbidity index, primary renal disease, smoking history, medication (ACEi/ARBs, diuretics, number of anti-HTN drugs, statins), BMI, and SBP. Model 3, model 2 + adjusted for hemoglobin, albumin, fasting glucose, HDL-C, TG, 25(OH) vitamin D, hs-CRP, GFR and spot urine ACR. Model 4, model 3 + adjusted for EF at the baseline. CI, confidence interval; HR, hazard ratio.*

### Sensitivity Analysis

After excluding the subjects with LEVE < 50%, both eLVH (adjusted HR 1.494, 95% CI 1.189–1.877) and cLVH (adjusted HR 1.286, 95% CI 1.006–1.643) were associated with increased risk of composite renal events, while concentric remodeling (adjusted HR 1.993, 95% CI 1.201–3.307) and cLVH (adjusted HR 2.220, 95% CI 1.329–3.706) were associated with all-cause mortality, demonstrating the association between LV geometry and study outcomes that is independent of LV systolic dysfunction (Table 4). After excluding the subjects with increased RWT, increased LVMI (i.e., eLVH) was robustly associated with increased risk of composite renal events (adjusted HR 1.588, 95% CI 1.261–2.001), but was not significant associated with all-cause mortality (Supplementary Table 4). After excluding the subjects with LVH, increased RWT (i.e., concentric remodeling) was still significantly associated with all-cause mortality (adjusted HR 1.993, 95% CI 1.197–3.368), but was not significant associated with increased risk of composite renal events (Supplementary Table 5).

**TABLE 4:** Cox regression analysis of LV geometry for primary outcomes in the subjects with EF ≥ 50%.

	LV geometry	Events, *n* (%)	Model 1		Model 2		Model 3		Model 4	
						
			HR (95% CIs)	*P*-value	HR (95% CIs)	*P*-value	HR (95% CIs)	*P*-value	HR (95% CIs)	*P*-value
Composite renal event	Normal	399 (31.0)	Reference		Reference		Reference		Reference	
	Concentric remodeling	93 (30.2)	1.062 (0.839, 1.344)	0.616	0.903 (0.717, 1.136)	0.383	1.012 (0.794, 1.29)	0.922	1.011 (0.794, 1.288)	0.929
	Eccentric hypertrophy	128 (50.2)	1.927 (1.559, 2.382)	<0.001	1.559 (1.256, 1.935)	<0.001	1.484 (1.181, 1.863)	<0.001	1.494 (1.189, 1.877)	<0.001
	Concentric hypertrophy	111 (43.0)	2.012 (1.605, 2.522)	<0.001	1.557 (1.249, 1.941)	<0.001	1.28 0(1.002, 1.636)	0.048	1.286 (1.006, 1.643)	0.044
All-cause mortality	Normal	90 (7.0)	Reference		Reference		Reference		Reference	
	Concentric remodeling	26 (8.4)	2.566 (1.583, 4.160)	<0.001	1.935 (1.207, 3.102)	0.006	1.942 (1.171, 3.219)	0.0101	1.993 (1.201, 3.307)	0.008
	Eccentric hypertrophy	23 (9.0)	1.252 (0.647, 2.420)	0.505	0.926 (0.492, 1.743)	0.811	0.823 (0.414, 1.637)	0.579	0.821 (0.413, 1.632)	0.573
	Concentric hypertrophy	31 (12.0)	4.791 (3.067, 7.484)	<0.001	2.620 (1.650, 4.159)	<0.001	2.161 (1.298, 3.597)	0.0031	2.220 (1.329, 3.706)	0.002

*Model 1, unadjusted model. Model 2, model 1 + adjusted for age, sex, Charlson comorbidity index, primary renal disease, smoking history, medication (ACEi/ARBs, diuretics, number of anti-HTN drugs, statins), BMI, and SBP. Model 3, model 2 + adjusted for hemoglobin, albumin, fasting glucose, HDL-C, TG, 25(OH) vitamin D, hs-CRP, GFR and spot urine ACR. Model 4, model 3 + adjusted for EF at the baseline. CI, confidence interval; HR, hazard ratio.*

### Subgroup Analysis

Subgroup analyses revealed that the association between LV geometry, especially eLVH, and the risk of composite renal events was more prominent in the subjects with BMI < 23 kg/m^2^ (*P* for interaction = 0.006) and eGFR ≥ 45 mL/min/1.73 m^2^ (*P* for interaction = 0.018) than in the subjects with BMI ≥ 23 kg/m^2^ and eGFR < 45 mL/min/1.73 m^2^ (Table 5), while age, sex, and spot urine ACR did not modify the association between LV geometry and the risk of composite renal events. None of age, sex, BMI, eGFR, and spot urine ACR modified the association between LV geometry and all-cause mortality (Supplementary Table 6).

**TABLE 5:** Cox regression analysis of LV geometry for composite renal event in various subgroups.

	LV geometry	Events, *n* (%)	Unadjusted HR (95% CIs)	*P* for interaction	Adjusted HR (95% CIs)	*P* for interaction
Age < 60 years	Normal	291 (30.8)	Reference	0.704	Reference	0.808
	Concentric remodeling	57 (32.9)	1.165 (0.877, 1.548)		1.034 (0.761, 1.405)	
	Eccentric hypertrophy	74 (48.7)	1.877 (1.454, 2.423)		1.612 (1.201, 2.164)	
	Concentric hypertrophy	49 (40.5)	1.849 (1.365, 2.503)		1.365 (0.960, 1.941)	
Age ≥ 60 years	Normal	111 (31.4)	Reference		Reference	
	Concentric remodeling	37 (27.2)	0.910 (0.627, 1.322)		0.958 (0.635, 1.444)	
	Eccentric hypertrophy	59 (49.2)	1.910 (1.392, 2.620)		1.339 (0.934, 1.919)	
	Concentric hypertrophy	63 (44.7)	1.981 (1.453, 2.701)		1.203 (0.838, 1.726)	
Male	Normal	255 (30.8)	Reference	0.442	Reference	0.447
	Concentric remodeling	68 (30.6)	1.071 (0.819, 1.399)		0.922 (0.690, 1.232)	
	Eccentric hypertrophy	57 (52.3)	2.298 (1.723, 3.063)		1.521 (1.085, 2.132)	
	Concentric hypertrophy	64 (42.1)	1.877 (1.426, 2.469)		1.078 (0.779, 1.490)	
Female	Normal	147 (31.2)	Reference		Reference	
	Concentric remodeling	26 (29.9)	1.037 (0.683, 1.574)		1.433 (0.902, 2.276)	
	Eccentric hypertrophy	76 (46.6)	1.736 (1.315, 2.290)		1.729 (1.243, 2.405)	
	Concentric hypertrophy	48 (43.6)	2.096 (1.511, 2.906)		1.882 (1.265, 2.801)	
BMI < 23 kg/m^2^	Normal	139 (30.0)	Reference	0.045	Reference	0.006
	Concentric remodeling	26 (31.0)	1.227 (0.807, 1.866)		0.924 (0.562, 1.519)	
	Eccentric hypertrophy	42 (54.5)	2.522 (1.784, 3.566)		2.030 (1.515, 3.501)	
	Concentric hypertrophy	29 (50.9)	3.036 (2.030, 4.540)		2.238 (1.330, 3.765)	
BMI ≥ 23 kg/m^2^	Normal	263 (31.5)	Reference		Reference	
	Concentric remodeling	68 (30.2)	0.997 (0.763, 1.301)		1.604 (0.797, 1.421)	
	Eccentric hypertrophy	91 (46.7)	1.692 (1.333, 2.147)		1.369 (1.039, 1.804)	
	Concentric hypertrophy	83 (40.5)	1.707 (1.333, 2.185)		1.127 (0.848, 1.498)	
eGFR ≥ 45 mL/min./1.73 m^2^	Normal	92 (12.7)	Reference	0.264	Reference	0.018
	Concentric remodeling	15 (10.6)	0.976 (0.565, 1.685)		1.067 (0.596, 1.911)	
	Eccentric hypertrophy	24 (26.1)	2.033 (1.297, 3.186)		3.135 (1.861, 5.280)	
	Concentric hypertrophy	9 (11.2)	1.070 (0.539, 2.122)		1.312 (0.575, 2.998)	
eGFR < 45 mL/min./1.73 m^2^	Normal	310 (54.1)	Reference		Reference	
	Concentric remodeling	79 (47.0)	0.841 (0.657, 1.077)		1.081 (0.825, 1.416)	
	Eccentric hypertrophy	109 (60.6)	1.366 (1.098, 1.700)		1.348 (1.049, 1.732)	
	Concentric hypertrophy	103 (56.6)	1.439 (1.151, 1.799)		1.159 (0.893, 1.504)	
Spot urine ACR < 300 mg/gCr	Normal	110 (17.1)	Reference	0.498	Reference	0.167
	Concentric remodeling	20 (13.3)	0.851 (0.528, 1.370)		0.730 (0.432, 1.232)	
	Eccentric hypertrophy	27 (29.0)	1.660 (1.089, 2.529)		1.297 (0.781, 2.154)	
	Concentric hypertrophy	15 (19.5)	1.267 (0.739, 2.174)		0.987 (0.544, 1.791)	
Spot urine ACR ≥ 300 mg/gCr	Normal	283 (46.7)	Reference		Reference	
	Concentric remodeling	72 (47.7)	1.122 (0.866, 1.453)		1.174 (0.910, 1.514)	
	Eccentric hypertrophy	102 (60.0)	1.697 (1.352, 2.129)		1.530 (1.220, 1.919)	
	Concentric hypertrophy	94 (53.4)	1.761 (1.393, 2.226)		1.317 (1.038, 1.671)	

*Models were adjusted for age, sex, Charlson comorbidity index, primary renal disease, smoking history, medication (ACEi/ARBs, diuretics, number of anti-HTN drugs, statins), BMI, SBP, hemoglobin, albumin, fasting glucose, HDL-C, TG, 25(OH) vitamin D, hs-CRP, GFR, spot urine ACR and EF at the baseline. ACR, albumin-to-creatinine ratio; CI, confidence interval; Cr, creatinine; eGFR, estimated glomerular filtration rate; HR, hazard ratio.*

## Discussion

In the present study, we unveiled that LV geometry is associated with adverse renal outcome and all-cause mortality in patients with pre-dialysis CKD. More specifically, concentric geometry and LVH were independently associated with all-cause mortality and adverse renal outcome, respectively. The association between LV geometry and adverse renal outcome was more prominent in the subjects with BMI < 23 kg/m^2^ and eGFR ≥ 45 mL/min/1.73 m^2^ than in the subjects with BMI ≥ 23 kg/m^2^ and eGFR < 45 mL/min/1.73 m^2^.

Only a few studies reported the association of LV geometry and outcomes in patients with pre-dialysis CKD (21, 24, 25). Although cLVH has been consistently associated with adverse CV (21, 24) or renal outcomes (21, 25), the prognostic significance of eLVH and concentric remodeling was less clear yet. A previous study reported that cLVH, but not eLVH, was associated with dialysis-free survival (25), where the analysis of concentric remodeling was even omitted. Another study reported both cLVH and eLVH are associated with adverse CV and renal outcomes in patients with CKD (21), though independent role of concentric remodeling or RWT was not proven. In contrast, to our best knowledge, the current study is the first report to demonstrate that eLVH is strongly associated with adverse renal outcome, and that concentric geometry or concentric remodeling alone is also independently associated with all-cause mortality, presenting more specific association of LV geometry patterns with study outcomes. This could be primarily attributed to substantially larger number of subjects included in the analysis and relatively longer duration of follow-up periods, rather than analytic bias. Therefore, the findings in the current study expand our understanding of the prognostic value of LV geometry in patients with pre-dialysis CKD.

In the present study, cLVH was associated with both adverse renal outcomes and all-cause mortality, which could be partially attributed to impaired microvascular function associated with high prevalence of DM among the subjects with cLVH (26, 27). On the other hand, one of the intriguing findings in the present study is a prominent contribution of eLVH to adverse renal outcomes, as eLVH, but not cLVH, was significantly associated with secondary outcomes (Supplementary Table 2), even though the subjects with cLVH were mostly associated with unfavorable baseline characteristics. It seems that the role of LV geometry pattern largely depends on the clinical contexts, as previous studied reported a significant association of eLVH with CV outcomes or all-cause mortality that is distinguished from cLVH (28, 29). Among the patients who underwent transcatheter aortic valve replacement, cLVH, especially mild cLVH, was independently associated with a decreased risk for mortality compared to normal geometry, while eLVH was associated with a 33% increased risk for mortality compared to cLVH (29). Similarly, eLVH was less responsive to ACEi treatment and was associated with a greater risk of adverse CV events compared with cLVH in patients on chronic hemodialysis (28). Moreover, complete correction of anemia was associated with reduced CV-event free survival specifically in patients with eLVH (24). Provided that cardiac remodeling begins as an adaptive process (16), it is speculated that a pathophysiology involved in pre-dialysis CKD may confer a distinct prognostic value on eLVH, although further studies are warranted to reveal the precise mechanism.

Currently, although losartan, an ARB, has shown a superiority to atenolol, a beta blocker, in the reversal of LVH, both agents were not different in the efficacy to reverse RWT (30). Most studies (31, 32) so far on pharmacologic interventions to reverse LV geometry are focusing on blood pressure lowering effect, not the class effect of antihypertensive agents. It should be further investigated, therefore, to determine the optimal regimen for antihypertensive drugs that leads to normalization of LV geometry.

It is of note that the association between LV geometry and the risk of composite renal events was more prominent in the subjects with eGFR ≥ 45 mL/min/1.73 m^2^ than in those with eGFR < 45 mL/min/1.73 m^2^. The early identification for the patients with high risk of CKD progression by echocardiographic determination of LV geometry may confer additional therapeutic benefits, as LVH is a potentially modifiable CV risk factor (2–4). Several trials have shown that intensive hemodialysis alleviates LVH among the patient with ESRD (33–35), while observational studies suggested that intensive hemodialysis may confer CV benefits (36). Accordingly, despite the lack of direct evidence that the regression of LVH improves the clinical outcomes in pre-dialysis CKD, it is expected that echocardiographic examination at early stages of CKD may help guide the intensive medical treatment to prevent or reverse LVH.

### Limitations

Some limitations are to be acknowledged in the present study. First, we are not able to confirm the casual relation between LV geometry and the study outcomes, because of the observational study design. Similarly, further studies are required to determine whether normalization of LV geometry improves renal outcome in patients with pre-dialysis CKD. Second, echocardiographic measurements were obtained from individual participating centers, and were not centralized with significant inter-observer variabilities in the measured parameters (Supplementary Table 7), whereas the multicenter nature of the current study is a strength. Third, as we did not measure right-side parameters, we are not able to assess the prognostic impact of pulmonary hypertension on adverse outcomes (37, 38). Fourth, as only ethnic Koreans were enrolled this cohort study, an extrapolation of the data to other populations requires precaution.

## Conclusion

In conclusion, we report that LV geometry is associated with adverse renal outcome and all-cause mortality in patients with pre-dialysis CKD. More specifically, concentric geometry and LVH are independently associated with all-cause mortality and adverse renal outcome, respectively. These results suggest that echocardiographic determination of LV geometry may help the early identification for the patients with high risk of CKD progression.

## Data Availability Statement

The raw data supporting the conclusions of this article will be made available by the authors, without undue reservation.

## Ethics Statement

The studies involving human participants were reviewed and approved by the study was conducted in accordance with the principles of the Declaration of Helsinki, and the study protocol was approved by the institutional review boards of participating centers, including at Seoul National University Hospital, Yonsei University Severance Hospital, Kangbuk Samsung Medical Center, Seoul St. Mary’s Hospital, Gil Hospital, Eulji General Hospital, Chonnam National University Hospital, and Pusan Paik Hospital. The patients/participants provided their written informed consent to participate in this study.

## Author Contributions

SS designed, helped in the data analysis and manuscript writing. SS, TO, and HC contributed to the conception of the study. SS and CK performed the data analyses and wrote the manuscript. EB, K-HO, JL, JJ, and K-BL collected the data. SM and SK helped perform the analysis with constructive discussions. All authors contributed to the article and approved the submitted version.

## Conflict of Interest

The authors declare that the research was conducted in the absence of any commercial or financial relationships that could be construed as a potential conflict of interest.

## Publisher’s Note

All claims expressed in this article are solely those of the authors and do not necessarily represent those of their affiliated organizations, or those of the publisher, the editors and the reviewers. Any product that may be evaluated in this article, or claim that may be made by its manufacturer, is not guaranteed or endorsed by the publisher.

## Footnotes

1
http://www.clinicaltrials.gov
