# Supplementary material for: Association Between Left Ventricular Geometry and Renal Outcomes in Patients With Chronic Kidney Disease: Findings From Korean Cohort Study for Outcomes in Patients With Chronic Kidney Disease Study
Source: Front Cardiovasc Med. 2022 Apr 18;9:848692. doi: 10.3389/fcvm.2022.848692 (PMC9058055; doi:10.3389/fcvm.2022.848692)
Supplement: Supplementary file 1 [file Data_Sheet_1.pdf]

# - Supplementary Material -

# Left ventricular geometry and renal outcomes in CKD: findings from KNOW-CKD study

Sang Heon Suh, M.D., Ph.D.[1], Tae Ryom Oh, M.D., Ph.D.[1], Hong Sang Choi, M.D., Ph.D.[1], Chang Seong Kim, M.D., Ph.D.[1], Eun Hui Bae, M.D., Ph.D.[1], Kook-Hwan Oh, M.D., Ph.D.[2], Joongyub Lee, M.D., Ph.D.[3], Ji Yong Jung, M.D., Ph.D.[4], Kyu-Beck Lee, M.D., Ph.D.[5], Seong Kwon Ma, M.D., Ph.D.[1], and Soo Wan Kim*, M.D., Ph.D.[1], on behalf of the Korean Cohort Study for Outcomes in Patients With Chronic Kidney Disease (KNOW-CKD) Investigators

[1]Department of Internal Medicine, Chonnam National University Medical School and Chonnam National University Hospital, Gwangju, Korea

[2]Department of Internal Medicine, Seoul National University Hospital, Seoul, Korea

[3]Department of Prevention and Management, School of Medicine, Inha University, Incheon, Republic of Korea

[4]Division of Nephrology, Department of Internal Medicine, Gachon University of Gil Medical Center, Incheon, Republic of Korea

[5]Department of Internal Medicine, Kangbuk Samsung Hospital, Sungkyunkwan University School of Medicine, Seoul, Republic of Korea

**Running title:** LV geometry and renal outcomes in CKD

*** Correspondence:**

*Soo Wan Kim, M.D., Ph.D., Department of Internal Medicine, Chonnam National University Medical School, 42 Jebongro, Gwangju 61469, Korea, Tel: +82-62-225-6271, Fax: +82-62-220-8578, Email: skimw@chonnam.ac.kr

**Table of Contents**

Table S1. Summary of echocardiographic findings of study participants by LV geometry

Table S2. Cox regression analysis of LV geometry for secondary outcomes

Table S3. Cox regression analysis of LVMI for primary outcomes

Table S4. Cox regression analysis of LVMI for primary outcomes in subjects without increased RWT

Table S5. Cox regression analysis of RWT for primary outcomes in subjects without LVH

Table S6. Cox regression analysis of LV geometry for all-cause mortality in various subgroups

Table S7. Summary of echocardiographic findings of study participants in the various participating centers

**Table S1. Summary of echocardiographic findings of study participants by LV geometry**

| | LV geometry | | | | |
|---|---|---|---|---|---|
| | Normal | Concentric remodeling | Eccentric hypertrophy | Concentric hypertrophy | *P* value |
| LVMI (g/m$^2$) | 81.556 ± 14.675 | 90.015 ± 13.678 | 118.630 ± 18.891 | 129.664 ± 26.124 | < 0.001 |
| RWT | 0.348 ± 0.043 | 0.465 ± 0.039 | 0.366 ± 0.039 | 0.484 ± 0.064 | < 0.001 |
| E/e’ | 9.124 ± 3.101 | 9.596 ± 3.072 | 11.951 ± 5.183 | 12.397 ± 4.989 | < 0.001 |
| LVEF (%) | 64.121 ± 5.908 | 64.963 ± 5.356 | 62.194 ± 7.784 | 64.987 ± 6.164 | < 0.001 |
| LAD (mm) | 36.803 ± 5.488 | 37.466 ± 5.545 | 40.344 ± 6.087 | 40.569 ± 6.173 | < 0.001 |
| RWMA | 26 (2.0) | 5 (1.6) | 16 (5.9) | 18 (6.8) | < 0.001 |
| Valve calcification | 76 (5.9) | 28 (9.1) | 33 (12.1) | 50 (19.0) | < 0.001 |
| PWT (mm) | 8.432 ± 1.062 | 10.340 ± 0.882 | 9.727 ± 1.009 | 11.783 ± 1.397 | < 0.001 |
| IVWT (mm) | 8.559 ± 1.233 | 10.175 ± 1.206 | 9.953 ± 1.185 | 11.877 ± 1.845 | < 0.001 |
| LVEDD (mm) | 48.649 ± 3.754 | 44.571 ± 3.687 | 53.377 ± 4.403 | 48.975 ± 4.095 | < 0.001 |
| LVESD (mm) | 30.423 ± 3.531 | 27.292 ± 3.345 | 34.216 ± 5.356 | 30.158 ± 4.319 | < 0.001 |

Note: Values for categorical variables are given as number (percentage); values for continuous variables, as mean ± standard deviation or median [interquartile range]. Abbreviations: E/e’, ratio of the early transmitral blood flow velocity to early diastolic velocity of the mitral annulus; IVWT, interventricular wall thickness; LAD, left atrium diameter; LVEDD, left ventricular end-diastolic diameter; LVEF, left ventricular ejection fraction; LVESD, left ventricular end-systolic diameter; LVMI, left ventricular mass index; PWT, posterior wall thickness; RMWA, regional wall motion abnormality.

**Table S2. Cox regression analysis of LV geometry for secondary outcomes**

| | LV geometry | Events, n (%) | Model 1 | | Model 2 | | Model 3 | | Model 4 | |
|---|---|---|---|---|---|---|---|---|---|---|
| | | | HR (95% CIs) | *P* value | HR (95% CIs) | *P* value | HR (95% CIs) | *P* value | HR (95% CIs) | *P* value |
| Decline of kidney function | Normal | 291 (22.4) | Reference | | Reference | | Reference | | Reference | |
| | Concentric remodeling | 66 (21.4) | 1.036 (0.784,1.370) | 0.803 | 0.922 (0.701,1.212) | 0.560 | 0.996 (0.747,1.327) | 0.977 | 1.001 (0.75,1.334) | 0.997 |
| | Eccentric hypertrophy | 92 (33.8) | 1.763 (1.377,2.257) | < 0.001 | 1.534 (1.194,1.972) | < 0.001 | 1.556 (1.189,2.036) | 0.001 | 1.535 (1.171,2.013) | 0.002 |
| | Concentric hypertrophy | 59 (22.5) | 1.239 (0.917,1.673) | 0.162 | 0.968 (0.723,1.298) | 0.830 | 0.78 (0.563,1.082) | 0.136 | 0.778 (0.561,1.079) | 0.132 |
| Onset of ESRD | Normal | 301 (23.2) | Reference | | Reference | | Reference | | Reference | |
| | Concentric remodeling | 74 (23.9) | 1.103 (0.843,1.442) | 0.474 | 0.916 (0.707,1.188) | 0.509 | 1.054 (0.798,1.394) | 0.710 | 1.061 (0.803,1.402) | 0.677 |
| | Eccentric hypertrophy | 100 (36.8) | 1.82 (1.427,2.321) | < 0.001 | 1.413 (1.106,1.805) | 0.006 | 1.373 (1.06,1.779) | 0.017 | 1.390 (1.072,1.802) | 0.013 |
| | Concentric hypertrophy | 98 (37.4) | 2.304 (1.804,2.942) | < 0.001 | 1.734 (1.364,2.203) | < 0.001 | 1.205 (0.92,1.579) | 0.175 | 1.206 (0.921,1.58) | 0.174 |

Note: Model 1, unadjusted model. Model 2, model 1 + adjusted for age, sex, Charlson comorbidity index, primary renal disease, smoking history, medication (ACEi/ARBs, diuretics, number of anti-HTN drugs, statins), BMI, and SBP. Model 3, model 2 + adjusted for hemoglobin, albumin, fasting glucose, HDL-C, TG, 25(OH) vitamin D, hs-CRP, GFR and spot urine ACR. Model 4, model 3 + adjusted for EF at the baseline. Abbreviations: CI, confidence interval; HR, hazard ratio.

**Table S3. Cox regression analysis of LVMI for primary outcomes**

| | LVMI | Events, n (%) | Model 1 | | Model 2 | | Model 3 | | Model 4 | |
|---|---|---|---|---|---|---|---|---|---|---|
| | | | HR (95% CIs) | *P* value | HR (95% CIs) | *P* value | HR (95% CIs) | *P* value | HR (95% CIs) | *P* value |
| Composite renal event | Normal | 496 (30.9) | Reference | | Reference | | Reference | | Reference | |
| | Increased | 245 (45.9) | 1.930 (1.640,2.272) | < 0.001 | 1.57 (1.329,1.854) | < 0.001 | 1.375 (1.148,1.647) | < 0.001 | 1.391 (1.161,1.667) | < 0.001 |
| All-cause mortality | Normal | 79 (4.9) | Reference | | Reference | | Reference | | Reference | |
| | Increased | 56 (10.5) | 2.403 (1.678,3.441) | < 0.001 | 1.542 (1.062,2.238) | 0.023 | 1.363 (0.907,2.046) | 0.136 | 1.325 (0.880,1.996) | 0.177 |

Note: Model 1, unadjusted model. Model 2, model 1 + adjusted for age, sex, Charlson comorbidity index, primary renal disease, smoking history, medication (ACEi/ARBs, diuretics, number of anti-HTN drugs, statins), BMI, and SBP. Model 3, model 2 + adjusted for hemoglobin, albumin, fasting glucose, HDL-C, TG, 25(OH) vitamin D, hs-CRP, GFR and spot urine ACR. Model 4, model 3 + adjusted for EF at the baseline. Abbreviations: CI, confidence interval; HR, hazard ratio.

**Table S4. Cox regression analysis of LVMI for primary outcomes in subjects without increased RWT**

| | LVMI | Events, n (%) | Model 1 | | Model 2 | | Model 3 | | Model 4 | |
|---|---|---|---|---|---|---|---|---|---|---|
| | | | HR (95% CIs) | *P* value | HR (95% CIs) | *P* value | HR (95% CIs) | *P* value | HR (95% CIs) | *P* value |
| Composite renal event | Normal | 402 (31.0) | Reference | | Reference | | Reference | | Reference | |
| | Increased | 133 (48.9) | 1.946 (1.580,2.397) | < 0.001 | 1.641 (1.319,2.042) | < 0.001 | 1.572 (1.250,1.978) | < 0.001 | 1.588 (1.261,2.001) | < 0.001 |
| All-cause mortality | Normal | 50 (3.9) | Reference | | Reference | | Reference | | Reference | |
| | Increased | 18 (6.6) | 1.707 (0.966,3.017) | 0.065 | 1.142 (0.641,2.036) | 0.652 | 1.010 (0.541,1.885) | 0.975 | 0.816 (0.420,1.584) | 0.548 |

Note: Model 1, unadjusted model. Model 2, model 1 + adjusted for age, sex, Charlson comorbidity index, primary renal disease, smoking history, medication (ACEi/ARBs, diuretics, number of anti-HTN drugs, statins), BMI, and SBP. Model 3, model 2 + adjusted for hemoglobin, albumin, fasting glucose, HDL-C, TG, 25(OH) vitamin D, hs-CRP, GFR and spot urine ACR. Model 4, model 3 + adjusted for EF at the baseline. Abbreviations: CI, confidence interval; HR, hazard ratio.

**Table S5. Cox regression analysis of RWT for primary outcomes in subjects without LVH**

| | RWT | Events, n (%) | Model 1 | | Model 2 | | Model 3 | | Model 4 | |
|---|---|---|---|---|---|---|---|---|---|---|
| | | | HR (95% CIs) | *P* value | HR (95% CIs) | *P* value | HR (95% CIs) | *P* value | HR (95% CIs) | *P* value |
| Composite renal event | Normal | 402 (31.0) | Reference | | Reference | | Reference | | Reference | |
| | Increased | 94 (30.4) | 1.077 (0.852,1.362) | 0.534 | 0.922 (0.731,1.162) | 0.492 | 1.034 (0.81,1.318) | 0.791 | 1.033 (0.810,1.317) | 0.793 |
| All-cause mortality | Normal | 50 (3.9) | Reference | | Reference | | Reference | | Reference | |
| | Increased | 29 (9.4) | 2.529 (1.563,4.092) | < 0.001 | 1.983 (1.225,3.211) | 0.005 | 1.968 (1.167,3.319) | 0.011 | 1.993 (1.179,3.368) | 0.010 |

Note: Model 1, unadjusted model. Model 2, model 1 + adjusted for age, sex, Charlson comorbidity index, primary renal disease, smoking history, medication (ACEi/ARBs, diuretics, number of anti-HTN drugs, statins), BMI, and SBP. Model 3, model 2 + adjusted for hemoglobin, albumin, fasting glucose, HDL-C, TG, 25(OH) vitamin D, hs-CRP, GFR and spot urine ACR. Model 4, model 3 + adjusted for EF at the baseline. Abbreviations: CI, confidence interval; HR, hazard ratio.

**Table S6. Cox regression analysis of LV geometry for all-cause mortality in various subgroups**

| | LV geometry | Events, n (%) | Unadjusted HR (95% CIs) | *P* for interaction | Adjusted HR (95% CIs) | *P* for interaction |
|---|---|---|---|---|---|---|
| Age < 60 years | Normal | 21 (2.2) | Reference | 0.706 | Reference | 0.856 |
| | Concentric remodeling | 8 (4.6) | 2.270 (1.005, 5.128) | | 1.956 (0.744, 5.144) | |
| | Eccentric hypertrophy | 4 (2.6) | 1.193 (0.410, 3.477) | | 0.953 (0.251, 3.611) | |
| | Concentric hypertrophy | 10 (8.3) | 4.533 (2.132, 9.639) | | 3.058 (1.158, 8.079) | |
| Age ≥ 60 years | Normal | 29 (8.2) | Reference | | Reference | |
| | Concentric remodeling | 21 (15.4) | 1.972 (1.124, 3.459) | | 1.977 (1.044, 3.744) | |
| | Eccentric hypertrophy | 14 (11.7) | 1.420 (0.750, 2.688) | | 1.114 (0.547, 2.269) | |
| | Concentric hypertrophy | 28 (19.9) | 2.751 (1.636, 4.626) | | 2.219 (1.137, 3.986) | |
| Male | Normal | 40 (4.8) | Reference | 0.328 | Reference | 0.418 |
| | Concentric remodeling | 21 (9.5) | 4.608 (1.818, 11.678) | | 5.282 (1.824, 15.296) | |
| | Eccentric hypertrophy | 12 (11.0) | 1.748 (0.635, 4.809) | | 1.382 (0.426, 4.482) | |
| | Concentric hypertrophy | 29 (19.1) | 4.745 (1.926, 11.690) | | 4.358 (1.348, 14.086) | |
| Female | Normal | 10 (2.1) | Reference | | Reference | |
| | Concentric remodeling | 8 (9.2) | 2.090 (1.230, 3.550) | | 1.454 (0.804, 2.629) | |
| | Eccentric hypertrophy | 6 (3.7) | 2.441 (1.280, 4.655) | | 0.980 (0.461, 2.085) | |
| | Concentric hypertrophy | 9 (8.2) | 4.676 (2.898, 7.545) | | 2.105 (1.169, 3.791) | |
| BMI < 23 kg/m$^2$ | Normal | 17 (3.7) | Reference | 0.092 | Reference | 0.320 |
| | Concentric remodeling | 13 (15.5) | 4.898 (2.376, 10.100) | | 3.314 (1.306, 8.409) | |
| | Eccentric hypertrophy | 3 (3.9) | 1.149 (0.336, 3.922) | | 0.400 (0.078, 2.046) | |
| | Concentric hypertrophy | 10 (17.5) | 5.949 (2.719, 13.016) | | 2.028 (0.694, 5.923) | |
| BMI ≥ 23 kg/m$^2$ | Normal | 33 (4.0) | Reference | | Reference | |
| | Concentric remodeling | 16 (7.1) | 1.884 (1.037, 3.424) | | 1.634 (0.843, 3.170) | |
| | Eccentric hypertrophy | 15 (7.7) | 1.946 (1.056, 3.583) | | 1.281 (0.626, 2.619) | |
| | Concentric hypertrophy | 28 (13.7) | 4.114 (2.485, 6.809) | | 2.784 (1.524, 5.087) | |
| eGFR ≥ 45 mL/min./1.73m$^2$ | Normal | 18 (2.5) | Reference | 0.150 | Reference | 0.434 |

| | | | | | | |
|---|---|---|---|---|---|---|
| | Concentric remodeling | 9 (6.4) | 2.943 (1.322, 6.554) | | 2.363 (0.926, 7.504) | |
| | Eccentric hypertrophy | 1 (1.1) | 0.406 (0.005, 3.004) | | 0.093 (0.008, 1.083) | |
| | Concentric hypertrophy | 3 (3.8) | 1.791 (0.527, 6.084) | | 1.575 (0.396, 6.260) | |
| eGFR < 45 mL/min./1.73m$^2$ | Normal | 32 (5.6) | Reference | | Reference | |
| | Concentric remodeling | 20 (11.9) | 2.155 (1.231, 3.773) | | 2.064 (1.117, 3.814) | |
| | Eccentric hypertrophy | 17 (9.4) | 1.788 (0.992, 3.220) | | 1.203 (0.613, 2.362) | |
| | Concentric hypertrophy | 35 (19.2) | 4.039 (2.499, 6.528) | | 2.287 (1.278, 4.092) | |
| Spot urine ACR < 300 mg/gCr | Normal | 25 (3.9) | Reference | 0.366 | Reference | 0.893 |
| | Concentric remodeling | 15 (10.0) | 2.879 (1.517, 5.462) | | 2.545 (1.168, 5.546) | |
| | Eccentric hypertrophy | 5 (5.4) | 1.266 (0.484, 3.308) | | 1.008 (0.342, 2.971) | |
| | Concentric hypertrophy | 7 (9.1) | 2.585 (1.118, 5.978) | | 1.796 (0.716, 4.500) | |
| Spot urine ACR ≥ 300 mg/gCr | Normal | 25 (4.1) | Reference | | Reference | |
| | Concentric remodeling | 13 (8.6) | 2.319 (1.186, 4.536) | | 2.361 (1.129, 4.937) | |
| | Eccentric hypertrophy | 12 (7.1) | 1.825 (0.930, 3.689) | | 1.277 (0.578, 2.819) | |
| | Concentric hypertrophy | 31 (17.6) | 5.094 (3.000, 8.650) | | 2.566 (1.334, 4.936) | |

Note: Models were adjusted for age, sex, Charlson comorbidity index, primary renal disease, smoking history, medication (ACEi/ARBs, diuretics, number of anti-HTN drugs, statins), BMI, SBP, hemoglobin, albumin, fasting glucose, HDL-C, TG, 25(OH) vitamin D, hs-CRP, GFR,spot urine ACR and EF at the baseline. Abbreviations: ACR, albumin-to-creatinine ratio; CI, confidence interval; Cr, creatinine; eGFR, estimated glomerular filtration rate; HR, hazard ratio.

**Table S7. Summary of echocardiographic findings of study participants in the various participating centers**

| | Participating centers | | | | | | | | | |
|---|---|---|---|---|---|---|---|---|---|---|
| | Hospital 1 | Hospital 2 | Hospital 3 | Hospital 4 | Hospital 5 | Hospital 6 | Hospital 7 | Hospital 8 | Hospital 9 | *P* value |
| LVMI (g/m$^{2.7}$) | 95.710 ± 20.821 | 85.355 ± 26.743 | 88.065 ± 22.409 | 88.608 ± 21.268 | 111.452 ± 26.800 | 101.986 ± 23.238 | 99.497 ± 26.189 | 86.995 ± 20.230 | 98.686 ± 28.556 | < 0.001 |
| RWT | 0.388 ± 0.051 | 0.345 ± 0.063 | 0.351 ± 0.058 | 0.336 ± 0.054 | 0.439 ± 0.078 | 0.403 ± 0.081 | 0.433 ± 0.078 | 0.381 ± 0.058 | 0.384 ± 0.067 | < 0.001 |
| E/e’ | 10.171 ± 3.401 | 9.915 ± 3.454 | 9.256 ± 2.985 | 10.988 ± 3.906 | 11.482 ± 4.212 | 9.497 ± 3.448 | 9.170 ± 3.645 | 9.352 ± 3.449 | 11.161 ± 5.454 | < 0.001 |
| LVEF (%) | 65.949 ± 6.940 | 66.113 ± 6.307 | 62.567 ± 5.394 | 67.082 ± 6.928 | 66.133 ± 5.444 | 62.322 ± 4.342 | 63.764 ± 5.223 | 61.959 ± 5.485 | 66.627 ± 6.800 | < 0.001 |
| LAD (mm) | 38.979 ± 6.154 | 38.353 ± 6.010 | 35.770 ± 4.815 | 39.366 ± 5.989 | 39.161 ± 5.315 | 37.079 ± 5.719 | 37.057 ± 5.927 | 38.430 ± 5.963 | 36.605 ± 5.821 | < 0.001 |
| RWMA | 1 (0.7) | 2 (1.6) | 5 (1.8) | 8 (4.5) | 11 (6.1) | 3 (2.5) | 18 (7.9) | 8 (1.3) | 9 (3.4) | 0.001 |
| Valve calcification | 5 (3.5) | 2 (1.6) | 2 (0.7) | 18 (10.1) | 3 (1.7) | 35 (29.2) | 19 (8.3) | 11 (1.7) | 92 (35.0) | < 0.001 |
| PWT (mm) | 9.412 ± 1.200 | 8.482 ± 1.644 | 8.669 ± 1.379 | 8.550 ± 1.318 | 10.633 ± 1.740 | 9.832 ± 1.673 | 10.079 ± 1.641 | 9.050 ± 1.305 | 9.430 ± 1.654 | < 0.001 |
| IVWT (mm) | 9.505 ± 1.280 | 8.724 ± 1.920 | 8.696 ± 1.497 | 8.343 ± 1.470 | 10.772 ± 1.842 | 9.855 ± 1.776 | 10.608 ± 1.840 | 9.046 ± 1.323 | 9.582 ± 1.721 | < 0.001 |
| LVEDD (mm) | 48.716 ± 4.406 | 49.263 ± 4.366 | 49.631 ± 4.151 | 51.184 ± 4.367 | 48.783 ± 4.584 | 49.159 ± 4.060 | 46.870 ± 4.547 | 47.809 ± 4.286 | 49.281 ± 4.549 | < 0.001 |
| LVESD (mm) | 30.862 ± 4.218 | 31.202 ± 3.706 | 31.277 ± 4.126 | 31.928 ± 4.257 | 30.844 ± 4.644 | 29.931 ± 4.362 | 28.449 ± 4.682 | 29.344 ± 3.568 | 32.103 ± 4.301 | < 0.001 |

Note: Values for categorical variables are given as number (percentage); values for continuous variables, as mean ± standard deviation or median [interquartile range]. Abbreviations: E/e’, ratio of the early transmitral blood flow velocity to early diastolic velocity of the mitral annulus; IVWT, interventricular wall thickness; LAD, left atrium diameter; LVEDD, left ventricular end-diastolic diameter; LVEF, left ventricular ejection fraction; LVESD, left ventricular end-

systolic diameter; LVMI, left ventricular mass index; PWT, posterior wall thickness; RMWA, regional wall motion abnormality.
